# Generative Pre-trained Transformer 4 analysis of cardiovascular magnetic resonance reports in suspected myocarditis: A multicenter study

**DOI:** 10.1016/j.jocmr.2024.101068

**Published:** 2024-07-28

**Authors:** Kenan Kaya, Carsten Gietzen, Robert Hahnfeldt, Maher Zoubi, Tilman Emrich, Moritz C. Halfmann, Malte Maria Sieren, Yannic Elser, Patrick Krumm, Jan M. Brendel, Konstantin Nikolaou, Nina Haag, Jan Borggrefe, Ricarda von Krüchten, Katharina Müller-Peltzer, Constantin Ehrengut, Timm Denecke, Andreas Hagendorff, Lukas Goertz, Roman J. Gertz, Alexander Christian Bunck, David Maintz, Thorsten Persigehl, Simon Lennartz, Julian A. Luetkens, Astha Jaiswal, Andra Iza Iuga, Lenhard Pennig, Jonathan Kottlors

**Affiliations:** a Institute for Diagnostic and Interventional Radiology, Faculty of Medicine and University Hospital Cologne, University of Cologne, Cologne, Germany; b Institute for Diagnostic and Interventional Radiology, Faculty of Medicine and University Hospital Bonn, University of Bonn, Bonn, Germany; c Department of Radiology, Diagnostic and Interventional Radiology, University of Tübingen, Tübingen, Germany; d Institute for Radiology, Neuroradiology and Nuclear Medicine Johannes Wesling University Hospital/Mühlenkreiskliniken, Bochum/Minden, Germany; e Department of Diagnostic and Interventional Radiology, University Medical Center of the Johannes-Gutenberg-University, Mainz, Germany; f Department of Radiology and Nuclear Medicine, UKSH, Campus Lübeck, Lübeck, Germany; g Institute of Interventional Radiology, UKSH, Campus Lübeck, Lübeck, Germany; h Department of Diagnostic and Interventional Radiology, Medical Center, Faculty of Medicine, University of Freiburg, Freiburg, Germany; i Department of Diagnostic and Interventional Radiology, University of Leipzig, Leipzig, Germany; j Department of Cardiology, University of Leipzig, Leipzig, Germany; k Division of Cardiovascular Imaging, Department of Radiology and Radiological Science, Medical University of South Carolina, Charleston, South Carolina, USA; l German Centre for Cardiovascular Research, Partner Site Rhine-Main, Mainz, Germany

**Keywords:** Cardiovascular magnetic resonance, Generative Pre-trained Transformer 4, Artificial intelligence, Large language models, Myocarditis

## Abstract

**Background:**

Diagnosing myocarditis relies on multimodal data, including cardiovascular magnetic resonance (CMR), clinical symptoms, and blood values. The correct interpretation and integration of CMR findings require radiological expertise and knowledge. We aimed to investigate the performance of Generative Pre-trained Transformer 4 (GPT-4), a large language model, for report-based medical decision-making in the context of cardiac MRI for suspected myocarditis.

**Methods:**

This retrospective study includes CMR reports from 396 patients with suspected myocarditis and eight centers, respectively. CMR reports and patient data including blood values, age, and further clinical information were provided to GPT-4 and radiologists with 1 (resident 1), 2 (resident 2), and 4 years (resident 3) of experience in CMR and knowledge of the 2018 Lake Louise Criteria. The final impression of the report regarding the radiological assessment of whether myocarditis is present or not was not provided. The performance of Generative pre-trained transformer 4 (GPT-4) and the human readers were compared to a consensus reading (two board-certified radiologists with 8 and 10 years of experience in CMR). Sensitivity, specificity, and accuracy were calculated.

**Results:**

GPT-4 yielded an accuracy of 83%, sensitivity of 90%, and specificity of 78%, which was comparable to the physician with 1 year of experience (R1: 86%, 90%, 84%, p = 0.14) and lower than that of more experienced physicians (R2: 89%, 86%, 91%, p = 0.007 and R3: 91%, 85%, 96%, p < 0.001). GPT-4 and human readers showed a higher diagnostic performance when results from T1- and T2-mapping sequences were part of the reports, for residents 1 and 3 with statistical significance (p = 0.004 and p = 0.02, respectively).

**Conclusion:**

GPT-4 yielded good accuracy for diagnosing myocarditis based on CMR reports in a large dataset from multiple centers and therefore holds the potential to serve as a diagnostic decision-supporting tool in this capacity, particularly for less experienced physicians. Further studies are required to explore the full potential and elucidate educational aspects of the integration of large language models in medical decision-making.

## Graphical abstract

**Figure:**
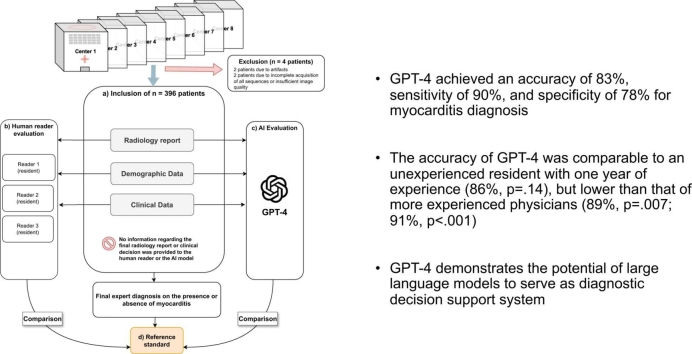

## 1 Introduction

Myocarditis represents an important cause of cardiac morbidity and mortality, leading to up to 20–40% of sudden cardiac deaths in patients younger than 40 years [1], [2]. Although an early and accurate diagnosis of myocarditis is mandatory to reduce the risk of progression, the correct diagnosis still poses a challenge in modern cardiology because of the variety of clinical representations and laboratory findings of myocarditis [3].

In this context, cardiac cardiovascular magnetic resonance (CMR) has evolved as a reliable non-invasive diagnostic tool in patients with suspected myocarditis [4]. The diagnosis of myocarditis using CMR requires a high level of radiological expertise and the ability to interpret various image characteristics in different sequences [5]. In 2009, the Lake Louise Criteria (LLC) were introduced for the diagnosis of myocarditis and were supplemented by quantitative mapping techniques in 2018 [6]. According to the revised LLC, diagnosis of myocarditis can be made when two main criteria are met: at least one T1-based criterion (increased myocardial T1 relaxation time, increased extracellular volume fraction, or positive late gadolinium enhancement [LGE]) and at least one T2-based criterion (increased myocardial T2 relaxation time or visual myocardial edema/increased T2 signal intensity ratio) [6]. While proficient cardiovascular imaging experts can make precise diagnoses of myocarditis, inexperienced radiologists exhibit a much lower level of accuracy in interpreting these distinct findings, leading to a higher likelihood of incorrect diagnoses [7].

Several studies have highlighted the feasibility and potential of utilizing artificial intelligence (AI) in medical decision-making, particularly in radiology [8], [9]. These studies predominantly concentrate on AI-based processing of visual information [10], [11], [12], [13]. However, textual information is the cornerstone for documentation and communication in radiology [14], [15]. Recent advances in large language models (LLM) have opened new opportunities for processing such text-based medical information [16], [17], [18], [19]. One LLM that has shown remarkable capabilities is the Generative Pre-trained Transformer (GPT-4), developed by OpenAI (San Francisco, California, USA) [20], [21]. GPT-4 is a fourth-generation deep learning model able to generate logical and semantically accurate responses to text-based input information and questions [22]. GPT-4 has been trained using a large collection of text data extracted from the World Wide Web and has been optimized for various language-related tasks, such as text completion, translation, and question answering. Experimental studies indicated that the predecessor model GPT-3 showed promising results in medical question-answering tasks, achieving passing scores in medical licensing examinations [23]. The use of LLMs, such as GPT-4, for immediate clinical decision-making based on radiology report texts, could provide several benefits, such as improved diagnostic accuracy and reduced variability in decision-making processes. With the ability to analyze and integrate text-based information, such models could aid in the interpretation of various image characteristics in different sequences as well as clinical information and laboratory results to identify cases of myocarditis.

The aim of this study was, therefore, to investigate the performance of GPT-4 for diagnosing myocarditis using different styles of CMR reports as well as clinical information and blood values from various study centers, and to compare its performance to radiologists with different levels of experience in cardiovascular imaging.

## 2 Materials and methods

### 2.1 Ethics

This retrospective study received ethical approval (23-1061-retro) and informed consent was waived due to the retrospective design of the investigation. Beyond the patient's aggregated age and sex, no personal information about the patient was transmitted to the GPT-4 model, especially no patient-identifying information was provided to the AI.

### 2.2 Data acquisition

Radiology departments of eight tertiary care medical centers were advised to each retrospectively screen their database and randomly select a total of 50 CMR reports of patients who were referred for suspected myocarditis. MRI examinations were performed according to respective in-house protocols for myocarditis. Furthermore, the patient’s age, gender, clinical symptoms of the patients, and a board-certified radiology report with a final diagnosis of the examination needed to be available. Centers were advised to provide the patient’s age, gender, and clinical symptoms. Additionally, laboratory results were provided by the centers, if available. Laboratory results included C-reactive protein (CRP), creatine kinase (CK), creatine kinase-MB (CK-MB), and high-sensitive cardiac troponin (Hs-cTn).

The following data were retrieved from the reports as baseline characteristics for the cohort: left ventricular ejection fraction (LVEF), LGE pattern (subepicardial, mid-myocardial, subendocardial, transmural, and absent LGE), mapping characteristics, additional image findings, and final diagnosis of cardiomyopathies.

### 2.3 Data preparation

The final impression of the report was extracted from the texts. Furthermore, reports were not included if significant artifacts or poor image quality was reported hindering the ability to make a certain diagnosis. After assessment regarding inclusion and exclusion criteria by the leading center (1), reports were excluded, if provided report data were insufficient or afflicted with errors such as missing text information.

The radiology report in [blinded for submission] language as well as clinical symptoms, laboratory values (if available), and aggregated patient age and gender were compiled into a text dataset in one Word document (Microsoft Office, Redmond, Washington) per patient.

**Fig. 1:**
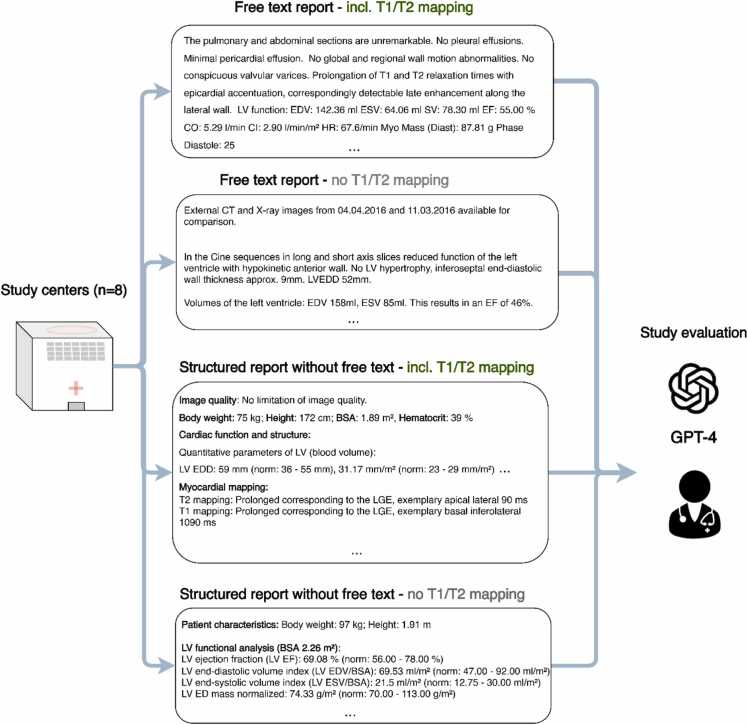
Exemplary styles of reports being included in this study: free text with T1/T2-mapping, free text without T1/T2-mapping, structured report with T1- and T2-mapping, and structured report without T1/T2-mapping. *LV* left ventricular, *EDV* end-diastolic volume, *ESV* end-systolic volume, *EF* ejection fraction, *CO* cardiac output, *CI* cardiac index, *HR* heart rate, *CT* computed tomography, *LV EDD* left ventricular end-diastolic diameter, *BSA* body surface area, *ED* end-diastole, *LGE* late gadolinium enhancement, *GPT-4* Generative Pre-trained Transformer 4.

Furthermore, subgroups were established based on the availability of (a) T1- and T2-mapping sequences, (b) laboratory values, and (c) structured reports (Fig. 1). Laboratory results were defined as available if all of the following were available: CRP, CK, CK-MB, and Hs-cTn (as shown in Graphical Abstract).

### 2.4 GPT-4

**Fig. 2:**
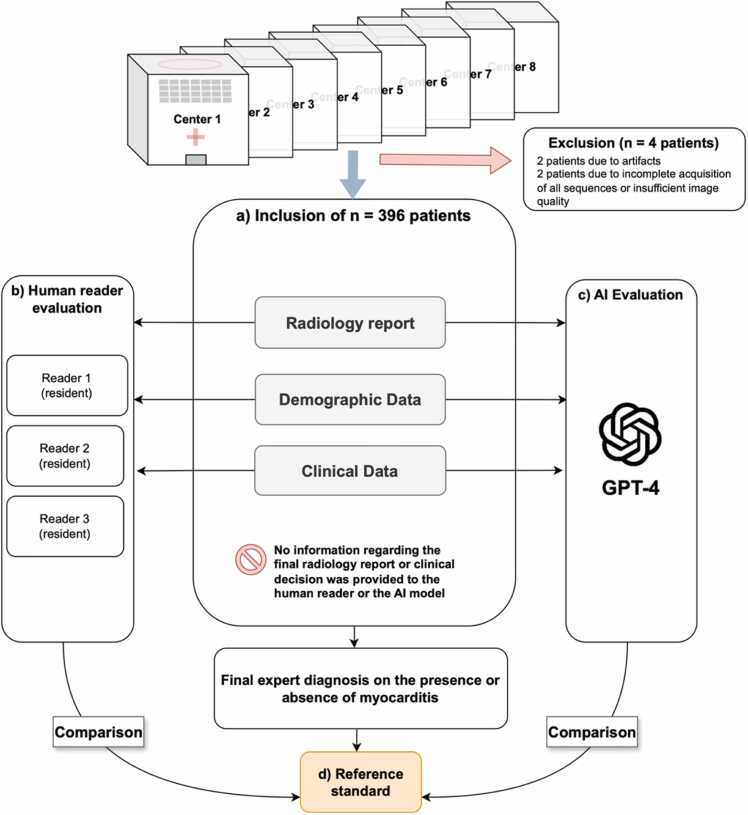
Workflow of the study design. The reference standard was established by the diagnosis of myocarditis based on the assessment of two board-certified radiologists with 8 and 10 years of experience in cardiovascular imaging, respectively. *GPT-4* Generative Pre-trained Transformer 4.

GPT-4 was accessed via OpenAIs (San Francisco, California, USA) web interface platform ChatGPT (https://chat.openai.com/) within the timeframe between March and July 2023 [21]. All text datasets were copied separately to the platform using one chat per text dataset. GPT-4 was prompted with evaluating each dataset using zero-shot prompting (Fig. 2).

### 2.5 Human reader

To provide a comparison of the performance of GPT-4 to human readers, the datasets were reviewed by three radiology residents with 1 (R.H.; resident 1), 2 (K.K.; resident 2), and 4 years (C.G.; resident 3) of experience in cardiovascular MRI. Knowledge of the 2018 LLC was a necessary precondition to serve as a human reader [6]. The evaluations were conducted independently and without a dedicated time limit (Fig. 2).

### 2.6 Prompting

The prompt for the human reader and GPT-4 was as follows:

*“Please decide on the presence or absence of myocarditis based on the radiological report, provided patient information and clinical parameters. Please respond with either ‘yes’ or ‘no’.”*

### 2.7 Reference standard

The reference standard was established by the diagnosis of myocarditis based on the assessment of two board-certified radiologists with 8 (L.P.) and 10 (A.I.) years of experience in CMR who reviewed the above-mentioned data and performed a consensus reading (Fig. 2). Consensus refers to a general agreement among the members of a particular group, each of whom has some level of autonomy in making decisions [24]. All human readers strictly adhered to the 2018 Lake Louise diagnostic criteria for myocarditis [6].

### 2.8 Statistical analysis

Statistical data analysis was performed using R version 4.4.1 (San Francisco, California, USA). The accuracy, precision, recall (sensitivity), F1 score, and specificity of the performance of GPT-4 and the human readers were calculated by comparing their evaluation to the reference standard and assessed using contingency tables. Dichotomous performance data were compared using McNemar's test or Pearson's chi-squared test. A p-value <0.05 was considered statistically significant. Figures were plotted using the ggplot2 package (Hadley Wickham, New Zealand). Continuous variables were reported as mean and standard deviation. Demographic characteristics were compared using the chi-squared test for categorical variables and the Mann-Whitney U test for continuous variables.

## 3 Results

### 3.1 Baseline characteristics

Of the available 400 reports, 4 patients were excluded due to artifacts or poor image quality and incomplete acquisition of all sequences (Fig. 2). Consequently, 396 patients were included for further analysis. Based on the final assessment in the reports, myocarditis was the most frequent diagnosis with 163 of 396 patients (41.2%), followed by ischemic cardiomyopathy with 23 of 396 patients (5.8%). Table 1 provides detailed results for the final diagnosis of CMR studies from the impressions of the individual reports by the respective centers. Regarding LGE, a subepicardial pattern was the most prevalent localization in 143 of 396 patients (36.1%), followed by mid-myocardial lesions. Table 2 lists the LGE pattern based on the reports. A mean LVEF of 54.9 ± 12.1% was observed.

**Table 1:** Final diagnosis of CMR based on the final diagnosis of the reports from the respective centers.

Final diagnosis	n = 396 patients
Non-ischemic cardiomyopathies	238/396 (60.1%)
Chemotherapy-induced toxicity	1/396 (0.3%)
Dilated cardiomyopathy	24/396 (6.1%)
Hypertrophic cardiomyopathy	12/396 (3.0%)
Non-compaction cardiomyopathy	1/396 (0.3%)
Myocarditis	163/396 (41.2%)
Pericarditis	23/396 (5.8%)
Sarcoidosis	3/396 (0.8%)
Takotsubo cardiomyopathy	7/396 (1.8%)
Ischemic cardiomyopathy	23/396 (5.8%)
Valvulopathy	6/396 (1.5%)
Uncertain findings	6/396 (1.5%)
No finding	143/396 (36.1%)

*CMR* cardiovascular magnetic resonance.

Table 1: Data are numbers (%) of 396 cases with their final diagnosis.

**Table 2:** LGE pattern based on the reports.

LGE localization	n = 396 patients
Subendocardial	24/396 (6.1%)
Mid-myocardial	78/396 (19.7%)
Subepicardial	143/396 (36.1%)
Transmural	35/396 (8.8%)
Absence of LGE	172/396 (43.3%)

*LGE* late gadolinium enhancement.

Table 2: Data are numbers (%) of 396 cases with their LGE localization in the text reports.

### 3.2 Assessment by the expert reader

According to the assessment by the expert readers (consensus), 171 of 396 patients (43.2%) showed myocarditis (group 1), whereas 225 of 396 patients (56.8%) did not (group 2). Patients in group 1 were significantly younger (38.6 ± 17.7 vs 44.4 ± 17.6 years; p = 0.001) and predominantly male (76.0% vs 56.9%; p < 0.001). Table 3 lists the demographic values for each center.

**Table 3:** Demographic characteristics of patients with myocarditis and without myocarditis.

		Myocarditis			No myocarditis		
	N	N	Age	Sex	N	Age	Sex
Center 1	50	27	34.1 ± 14.6 (18, 62)	5 F, 22 M	23	42.9 ± 19.0 (18, 73)	12 F, 11 M
Center 2	50	25	42.5 ± 21.6 (16, 83)	8 F, 17 M	25	52 ± 21.3 (9, 85)	14 F, 11 M
Center 3	50	18	51.7 ± 16.7 (19, 75)	6 F, 12 M	32	52 ± 18.2 (26, 86)	7 F, 25 M
Center 4	50	19	42.4 ± 15.2 (24, 71)	4 F, 15 M	31	38.9 ± 12.9 (18, 62)	14 F, 17 M
Center 5	50	21	35 ± 15.8 (18, 77)	7 F, 14 M	29	37.7 ± 11.0 (20, 60)	10 F, 19 M
Center 6	48	16	29.8 ± 16.3 (3, 61)	4 F, 12 M	32	42.3 ± 20.5 (11, 84)	12 F, 20 M
Center 7	48	24	36.8 ± 18.4 (15, 77)	2 F, 22 M	24	42.3 ± 15.7 (16, 77)	13 F, 11 M
Center 8	50	21	37.8 ± 15.5 (20, 80)	5 F, 16 M	29	47.2 ± 16.4 (19, 83)	15 F, 14 M
All centers	396	171	38.6 ± 17.7 (3, 83)	41 F, 130 M	225	44.4 ± 17.6 (9, 86)	97 F, 128 M

*N* number (ages are reported as means ± standard)*, F* female*, M* male*.*

Values in parentheses are ranges.

Table 3: Data of the respective centers with their means ± standard deviation of age.

### 3.3 Performance of GPT-4

Compared to the expert reading, GPT-4 had an accuracy of 83%, specificity of 78%, and sensitivity of 90%. Table 4 provides detailed results for the performance of GPT-4.

**Table 4:** Performance of GPT-4 and radiology residents with 1 (resident 1), 2 (resident 2), and 4 years (resident 3) of experience compared to the reference standard.

	Accuracy	Precision	Recall (sensitivity)	F1 score	Specificity
GPT-4	0.83 (330/396)	0.76 (154/203)	0.90 (154/171)	0.82 (308/374)	0.78 (176/225)
Resident 1	0.86 (342/396) (p = 0.14)	0.81 (154/191)	0.90 (154/171)	0.85 (308/362)	0.84 (188/225)
Resident 2	0.89 (352/396) (**p** **=** **0.007**)	0.88 (147/167)	0.86 (147/171)	0.87 (294/338)	0.91 (205/225)
Resident 3	0.91 (361/396) (**p** **<** **0.001**)	0.94 (146/156)	0.85 (146/171)	0.89 (292/327)	0.96 (215/225)

*GPT-4* Generative Pre-trained Transformer 4*, F1 score* measurement of predictive performance*.*

The difference in accuracy between GPT-4 and radiology residents is shown as p values, bold indicates statistical significance.

The re-test-evaluation within the timeframe between July and August 2023 showed a 100% concordance between the results of the first and second GPT-4 evaluations.

### 3.4 Performance of the radiologists

**Fig. 3:**
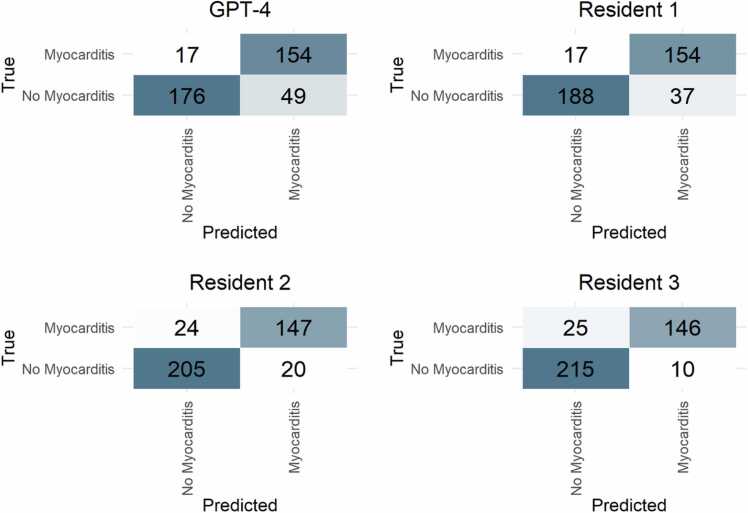
Confusion matrices for performance of GPT-4, residents 1, 2, and 3 compared to the reference standard. GPT-4: Generative Pre-trained Transformer 4.

Compared to the expert reading, resident 1 showed an accuracy of 86%, resident 2 of 89%, and resident 3 of 91%. The performance of resident 1 was comparable to GPT-4 (p = 0.14) whereas the more experienced readers showed superior results (p = 0.007 and p < 0.001, respectively). Table 4 gives detailed results for the performance of the radiologists. The experienced radiology residents (residents 2 and 3) showed no significant difference in accuracy (p = 0.22). Fig. 3 depicts confusion matrices for the performance of GPT-4 and radiologists compared to the reference standard.

**Fig. 4:**
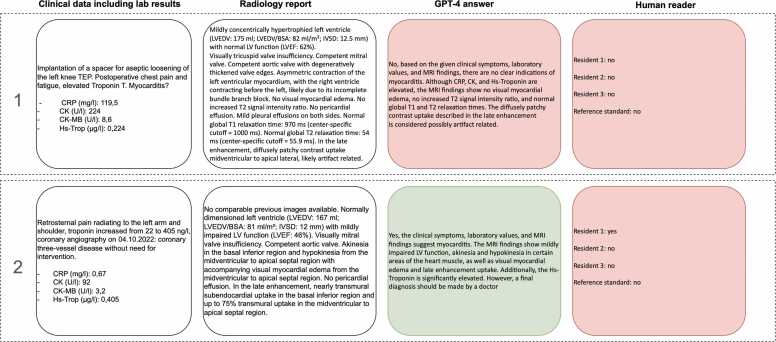
Proofreading examples by GPT-4 based on the given clinical data, laboratory values, and the radiology report compared to the assessment of the human readers. *CRP* C-reactive protein, *CK* creatine kinase, *CK-MB* creatine kinase-MB, *Hs-Trop* high sensitive troponin. *IVSD* interventricular septum thickness. *LV* left ventricular, *EDV* end-diastolic volume, *EF* ejection fraction, *BSA* body surface area, *GPT-4* Generative Pre-trained Transformer 4, *CMR* cardiovascular magnetic resonance .

Fig. 4 provides two examples of text-based analysis created by GPT-4 and the final assessment of GPT-4 and the human readers.

### 3.5 Subgroup analysis

#### 3.5.1 Distribution of the subgroups

T1- and T2-mapping was available from 250 of 396 patients (63.1%), laboratory values from 166 of 396 patients (41.9%), and structured reports from 246 of 396 patients (62.1%). The distribution of subgroups is presented in Table 5, as assessed by the expert reader.

**Table 5:** Distribution of the subgroups according to cases with myocarditis, no myocarditis, and all cases regarding the availability (yes = available, no = not available) of mapping, laboratory values, and structured reports.

	Mapping		Laboratory values		Structured reports	
	No	Yes	No	Yes	No	Yes
Myocarditis	58/396 (14.6%)	113/396 (28.5%)	74/396 (18.7%)	97/396 (24.5%)	65/396 (16.4%)	106/396 (26.8%)
No myocarditis	88/396 (22.2%)	137/396 (34.6%)	156/396 (39.4%)	69/396 (17.4%)	85/396 (21.5%)	140/396 (35.4%)
Total	146/396 (36.9%)	250/396 (63.1%)	230/396 (58.1%)	166/396 (41.9%)	150/396 (37.9%)	246/396 (62.1%)

A structured report is a method of clinical documentation in standardized formats.

##### 3.5.1.1 Subgroup laboratory values

For the subgroup according to laboratory values available vs unavailable, Table 6 gives detailed results. For GPT-4 (accuracy 85% vs 81%), resident 1 (accuracy 84% vs 89%), resident 2 (accuracy 87% vs 91%), and resident 3 (accuracy 89% vs 94%), no statistically significant differences in performance were observed between the subgroups with or without available laboratory values (p = 0.44, p = 0.22, p = 0.34, and p = 0.13, respectively).

**Table 6:** Performance of GPT-4 and radiologists with 1 (resident 1), 2 (resident 2), and 4 years (resident 3) of experience regarding the availability (yes = available, no = not available) of laboratory values.

	Accuracy		Precision		Recall (sensitivity)		F1 score		Specificity	
Laboratory values	No	Yes	No	Yes	No	Yes	No	Yes	No	Yes
GPT-4	0.85 (195/230)	0.81 (135/166) (p = 0.44)	0.71 (67/95)	0.81 (87/108)	0.91 (67/74)	0.90 (87/97)	0.79 (134/169)	0.85 (174/205)	0.82 (128/156)	0.70 (48/69)
Resident 1	0.84 (194/230)	0.89 (148/166) (p = 0.22)	0.72 (63/88)	0.88 (91/103)	0.85 (63/74)	0.94 (91/97)	0.78 (126/162)	0.91 (182/200)	0.84 (131/156)	0.83 (57/69)
Resident 2	0.87 (201/230)	0.91 (151/166) (p = 0.34)	0.81 (59/73)	0.94 (88/94)	0.79 (59/74)	0.91 (88/97)	0.80 (118/147)	0.92 (176/191)	0.91 (142/156)	0.91 (63/69)
Resident 3	0.89 (205/230)	0.94 (156/166) (p = 0.13)	0.89 (56/63)	0.97 (90/93)	0.76 (56/74)	0.93 (90/97)	0.82 (112/137)	0.95 (180/190)	0.96 (149/156)	0.96 (66/69)

*GPT-4* Generative Pre-trained Transformer 4*, F1 score* measurement of predictive performance*.*

The difference in accuracy with and without the availability of laboratory values is shown as p values.

##### 3.5.1.2 Subgroup T1- and T2-mapping sequences

GPT-4 (accuracy 79% vs 86%) as well as all residents, resident 1 (accuracy 79% vs 90%), resident 2 (accuracy 85% vs 91%), and resident 3 (accuracy 86% vs 94%), had an improved performance when mapping sequences were part of the reports. Residents 1 and 3 showed significant differences in their diagnostic performance regarding the availability of mapping sequences (p = 0.004 and p = 0.02, respectively). Table 7 summarizes the results of the mapping subgroup analysis.

**Table 7:** Performance of GPT-4 and radiologists with 1 (resident 1), 2 (resident 2), and 4 years (resident 3) of experience regarding the availability (yes = available, no = not available) of mapping.

	Accuracy		Precision		Recall (sensitivity)		F1 score		Specificity	
Mapping	No	Yes	No	Yes	No	Yes	No	Yes	No	Yes
GPT−4	0.79 (115/146)	0.86 (215/250) (p = 0.08)	0.67 (53/79)	0.81 (101/124)	0.91 (53/58)	0.89 (101/113)	0.77 (106/137)	0.85 (202/237)	0.70 (62/88)	0.83 (114/137)
Resident 1	0.79 (116/146)	0.90 (226/250) (**p** **=** **0.004**)	0.69 (50/72)	0.87 (104/119)	0.86 (50/58)	0.92 (104/113)	0.77 (100/130)	0.90 (208/232)	0.75 (66/88)	0.89 (122/137)
Resident 2	0.85 (124/146)	0.91 (228/250) (p = 0.08)	0.81 (47/58)	0.92 (100/109)	0.81 (47/58)	0.88 (100/113)	0.81 (94/116)	0.90 (200/222)	0.88 (77/88)	0.93 (128/137)
Resident 3	0.86 (126/146)	0.94 (235/250) (**p** **=** **0.02**)	0.87 (45/52)	0.97 (101/104)	0.78 (45/58)	0.89 (101/113)	0.82 (90/110)	0.93 (202/217)	0.92 (81/88)	0.98 (134/137)

*GPT-4* Generative Pre-trained Transformer 4*, F1 score* measurement of predictive performance*.*

The difference in accuracy with and without the mapping sequences is shown as p values, bold indicates statistical significance.

##### 3.5.1.3 Subgroup structured report

For GPT-4 (accuracy 81% vs. 85%), Resident 1 (accuracy 86% vs. 87%), Resident 2 (accuracy 93% vs. 87%), and Resident 3 (accuracy 91% vs. 91%), no significant differences were observed between structured and free-text radiological reports (p = 0.49, p = 0.99, p = 0.09, and p > 0.99, respectively). Please refer to Table 8 for detailed results.

**Table 8:** Performance of GPT-4 and radiologists with 1 (resident 1), 2 (resident 2), and 4 years (resident 3) of experience regarding the availability of structured report (yes = structured report, no = free-text report).

	Accuracy		Precision		Recall (sensitivity)		F1 score		Specificity	
Structured report	No	Yes	No	Yes	No	Yes	No	Yes	No	Yes
GPT-4	0.81 (122/150)	0.85 (208/246) (p = 0.49)	0.75 (55/73)	0.76 (99/130)	0.85 (55/65)	0.93 (99/106)	0.80 (110/138)	0.84 (198/236)	0.79 (67/85)	0.78 (109/140)
Resident 1	0.86 (129/150)	0.87 (213/246) (p = 0.99)	0.80 (59/74)	0.81 (95/117)	0.91 (59/65)	0.90 (95/106)	0.85 (118/139)	0.85 (190/223)	0.82 (70/85)	0.84 (118/140)
Resident 2	0.93 (139/150)	0.87 (213/246) (p = 0.09)	0.92 (59/64)	0.85 (88/103)	0.91 (59/65)	0.83 (88/106)	0.91 (118/129)	0.84 (176/209)	0.94 (80/85)	0.89 (125/140)
Resident 3	0.91 (137/150)	0.91 (224/246) (p > 0.99)	0.96 (54/56)	0.92 (92/100)	0.83 (54/65)	0.87 (92/106)	0.89 (108/121)	0.89 (184/206)	0.98 (83/85)	0.94 (132/140)

*GPT-4* Generative Pre-trained Transformer 4*, F1 score* measurement of predictive performance*.*

The difference in accuracy with and without structured reports is shown as p values.

## 4 Discussion

In this study, AI-assisted diagnosis of myocarditis solely based on CMR reports, laboratory results, and clinical information using GPT-4 was compared to the assessment of radiology residents with different levels of experience. Of note, neither the human readers nor GPT-4 had access to any imaging data. Using a consensus reading of two CMR experts as the reference standard, GPT-4 achieved a sufficient diagnostic performance, which was comparable to a first-year resident. While the availability of laboratory values showed a lower accuracy for GPT-4 based diagnosis, structured reports and available mapping sequences improved its diagnostic performance, albeit without yielding statistical significance.

The integration of diverse data sources has become increasingly important in the medical field. In this context, AI is playing a crucial role in supporting decision-making by assisting in the analysis of complex medical data and improving treatment planning [25], [26], [27]. Recently, GPT-4 has been widely recognized for its exceptional proficiency in assessing textual information, representing a major stride forward in natural language processing technology [21]. In a recently published study, GPT-4 succeeded in presenting complex medical findings in a simplified and understandable way for laypersons [28]. In other previous studies, GPT-4 has already shown its potential for applications in radiology. In this context, GPT-4 was able to provide assistance in the radiological workflow by enabling automated determination of radiologic study and protocol based on request forms [15], standardizing radiology reports [29], detecting errors in radiology reports [30], and transforming of free-text reports into structured reporting [31]. Furthermore, GPT-4 is capable of giving diagnostic support by providing accurate differential diagnosis of imaging patterns [14]. However, its performance to provide a final diagnosis is unknown.

The present study evaluates a new approach that utilizes GPT-4 as a text-processing AI model to aid in the decision-making process for the diagnosis of myocarditis. Furthermore, demographic data and clinical symptoms as well as laboratory values, if available, were provided to GPT-4 to reflect the real-world clinical scenario for the assessment of the presence of myocarditis.

Based on these findings, GPT-4 showed potential as an auxiliary tool for text-based diagnosis of myocarditis for inexperienced readers by yielding comparable accuracy. However, its performance was inferior to experienced readers, who showed a higher diagnostic accuracy. Of note, the availability of T1- and T2-mapping sequences as part of the reports improved the diagnostic performance of GPT-4 and of the human readers, for first- and fourth-year residents with statistical significance. These findings underline the necessity of mapping sequences for myocarditis diagnosis as indicated in previous studies comparing the original and 2018 LLC, which showed a higher diagnostic performance when implementing mapping sequences [32], [33]. Interestingly, GPT-4 showed a lower specificity when laboratory values were available potentially due to increased cardiac biomarkers not associated with myocarditis misleading the LLM into a wrong diagnosis, indicating necessary improvement of GPT-4 in the future. Despite not yielding statistical significance, GPT-4 had a higher diagnostic performance regarding the diagnosis of myocarditis when assessing structured reports. These findings emphasize the usefulness of structured reporting in radiology, leading to enhanced communication and facilitating collaboration among physicians [34].

Previous studies investigating the usefulness of GPT-4 in radiology mainly focused on data from a single center [14], [15], [18], [19], [31]. However, LLMs tend to show dependency on textual information and the language style of text information reports [35]. Furthermore, as shown in this study, there is a large variance in study protocols for CMR in suspected myocarditis. To this end, we decided to conduct the present study as a multi-center investigation by incorporating data sets from eight different institutions, including different styles of reporting and study protocols. Consequently, the present study gives insight into the real-world application of mapping sequences for suspected myocarditis 5 years after the introduction of the 2018 LLC with a third of examinations still being performed without the acquisition of mapping sequences [6]. Furthermore, despite not including image data, this work highlights the potential of incorporating the clinical setting (symptoms, laboratory results) for the final radiological assessment in terms of AI-supported combined diagnostics.

## 5 Limitations

The aforementioned strengths of this study are offset by some limitations, mostly related to the AI model itself. AI-based aspects, e.g. GPT-4, are considered language models that merely provide information but are not capable of critically questioning, understanding, and interpreting facts [18], [19]. Another limitation is due to the uncertain sources of the GPT-4s training dataset. This problem can lead to inconsistent and contradictory results. Future research should focus on LLMs with built-in capabilities to transparently disclose the exact sources or guidelines underlying their decision-making processes enabling the verification and critical evaluation of these references. Furthermore, the restricted access of GPT-4, potentially requiring the sharing of sensitive data with third parties, represents an additional limitation of the model. In contrast, competing models, e.g. Large Language Model Meta AI, Meta Platforms, Menlo Park, California, USA (LLaMA) [36], offer hospitals the potential to be applied within their infrastructure, abolishing the necessity to transfer data to external servers. Furthermore, the retrospective design and the binary diagnostic approach have to be regarded as limitations of this study since the latter does not reflect a real-world scenario with radiology reports occasionally including differential diagnosis. The chosen dichotomous yes or no approach regarding the presence of myocarditis most likely led to a selection bias potentially influencing the results. As in every study investigating AI-based diagnostic tasks, the chosen reference standard can be seen as a limitation of the study design. Despite implementing a consensus reading by two experts in cardiovascular imaging and given that the diagnostic criteria for myocarditis in terms of the 2018 LLC are somewhat straightforward, there still was a slight disagreement in 2% of cases between the official reports and the consensus reading, underlining the complexity of correct text-based interpretation. As another possible limitation of the study design, T1- and T2-mapping as well as an LGE may be described in ways that are suggestive of myocarditis, thus biasing GPT-4 and human readers.

In conclusion, this proof-of-concept study indicates the potential use of GPT-4 to assist radiology residents and radiologists inexperienced in cardiovascular imaging in diagnostic tasks, assuming the information in the body of the report is correct. However, future research, improvements, and specifications of LLMs are required to improve diagnostic performance and serve as a daily support or training tool.

## Funding

This work was partly funded by NUM 2 (Netzwerk Universitätsmedizin, Berlin, Germany) (FKZ: 01KX2121).

## Author contributions

**Andreas Hagendorff:** Investigation, Data curation. **Lukas Goertz:** Methodology, Data curation. **Roman J. Gertz:** Data curation. **Alexander Christian Bunck:** Project administration. **David Maintz:** Project administration. **Thorsten Persigehl:** Project administration. **Kenan Kaya:** Writing – original draft, Investigation, Conceptualization. **Jan Borggrefe:** Investigation, Data curation. **Ricarda von Krüchten:** Data curation. **Katharina Müller-Peltzer:** Data curation. **Constantin Ehrengut:** Data curation. **Timm Denecke:** Data curation. **Jonathan Kottlors:** Writing – original draft, Investigation, Conceptualization. **Yannic Elser:** Data curation. **Patrick Krumm:** Investigation, Data curation. **Jan M. Brendel:** Data curation. **Konstantin Nikolaou:** Investigation, Data curation. **Nina Haag:** Data curation. **Simon Lennartz:** Visualization, Supervision, Project administration. **Carsten Gietzen:** Writing – original draft, Investigation, Conceptualization. **Julian A. Luetkens:** Supervision, Project administration. **Robert Hahnfeldt:** Data curation. **Astha Jaiswal:** Visualization, Validation, Project administration, Methodology. **Maher Zoubi:** Data curation. **Lenhard Pennig:** Writing – original draft, Investigation, Conceptualization. **Tilman Emrich:** Investigation, Data curation. **Moritz C. Halfmann:** Investigation, Data curation. **Malte Maria Sieren:** Data curation. **Andra Iza Iuga**: Investiagation, Conceptualization.

## Declaration of competing interests

The authors declare the following financial interests/personal relationships which may be considered as potential competing interests: David Maintz received speaker’s honoraria from Philips Healthcare. Jan Borggrefe received speaker’s honoraria from Siemens Healthineers. Simon Lennartz is a member of Editorial Board of Radiology and a Senior Deputy Editor of Radiology in Training. Otherwise, the authors declare no conflicts of interest and had full control over all data, and guarantee correctness.
